# Retraction: Creation of ultrasound and temperature-triggered bubble liposomes from economical precursors to enhance the therapeutic efficacy of curcumin in cancer cells

**DOI:** 10.1039/c8ra90095g

**Published:** 2018-12-03

**Authors:** Andrew Shore

**Affiliations:** Royal Society of Chemistry Thomas Graham House, Science Park, Milton Road Cambridge CB4 0WF UK advances-rsc@rsc.org

## Abstract

Retraction of ‘Creation of ultrasound and temperature-triggered bubble liposomes from economical precursors to enhance the therapeutic efficacy of curcumin in cancer cells’ by Santanu Patra *et al.*, *RSC Adv.*, 2016, **6**, 85473–85485.

The Royal Society of Chemistry hereby wholly retracts this *RSC Advances* article due to concerns with the reliability of the data in the published article.

The SEM image in Fig. 1B and confocal laser scanning fluorescence images in Fig. 6A have been used in another publication, but have been presented as different materials.^1^

The critical aggregation concentration data presented in Fig. S12 duplicates data presented in other publications, but was reported as different materials.^1–3^

The zeta potential plot data presented in Fig. S13 duplicates data presented in other publications, but was reported as different materials.^3–6^

Given the number and significance of the concerns, the validity of the data and, therefore, the conclusions presented in this paper are no longer reliable.

The Royal Society of Chemistry apologises for the fact that these concerns were not identified during the peer review process.

Santanu Patra, Rashmi Madhuri and Prashant K. Sharma oppose the retraction. Ekta Roy was contacted but did not respond.

Signed: Andrew Shore, Executive Editor, *RSC Advances*.

Date: 23^rd^ November 2018.

## Supplementary Material
